# Detection of torque teno virus (TTV) in domestic village chickens in Iran

**Published:** 2013

**Authors:** Majid Bouzari, Nima Shaykh Baygloo

**Affiliations:** *Department of Biology, Faculty of Science, University of Isfahan, Isfahan, Iran.*

**Keywords:** Domestic village chickens, Iran, PCR, Torque Teno Virus (TTV)

## Abstract

Torque teno virus (TTV) is prevalent worldwide and has been extensively studied in human and some wild and domestic animals. As the studies on TTV in chickens was rare and there was no information about the infection of domestic village chickens with TTV and also structural resemblance of this virus to chicken anemia virus, the frequency of the infection in domestic village chickens in different villages in Isfahan (Iran) was investigated. Sera were collected from 50 chickens. Viral DNA was extracted and subjected to polymerase chain reaction (PCR) using the previously described T801 and T935 primers that were used for amplification of a highly conserved non-coding region (UTR) of the viral genome in a single round of PCR and Set B primers of conserved region in a nested PCR reaction. Using T801 and T835 primers TTV or viruses of TTV family were detected in 16 out of 50 sera tested (32%). Fourteen out of the same 50 sera (28%) were positive for TTV using Set B primers. Totally 20 sera were positive using both primers (40%). Ten sera were detected with both sets of primers, six sera with T801 and T935 primers and only four sera were positive using Set B primers for TTV. Different patterns of the detection of the virus with the two different sets of primers suggests the possibility of the presence of different genotypes of TTV in domestic village chickens and the possibility of the transmission of the virus from human to village chickens and vice versa. This necessitates further investigations.

## Introduction

Torque teno virus (TTV) is an unenveloped virus with a single-stranded, negative polarity, circular DNA genome that was initially recovered from the serum of a Japanese patient with post-transfusion hepatitis of unknown etiology in 1997.^1^^,^^2^ After its discovery, extensive studies on its prevalence in different disease conditions and healthy individuals have been undertaken in different countries including Iran. Using primers designed for conserved non-coding region of the genome of the virus, high prevalence of up to 100% of the virus is reported in the sera and plasma samples. ^3^^-^^6^ Torque teno virus (TTV) together with SEN virus (SENV), Torque teno mini virus (TTMV) or TTV like mini virus (TLMV), Small anellovirus (SAV), animal Torque teno viruses and other TTV like viruses are classified into Anelloviridae family.^7^ Due to high sequence homology of the Anelloviruses, primers designed for detection of each of these viruses can detect the others. For example T801 and T935 primers designed for detection of the prevalence of TTV can also detect SEN virus.^6^ TTV is a very stable virus and has been detected in a variety of clinical samples including blood, liver, bile, cervical swabs, saliva, semen, throat swabs, infant cord blood, amniotic fluid, breast milk, feces, hair and skin.^8^^-^^18^ Although higher viral load in patients with severe idiopathic inflammatory myopathies, cancer and lupus and also active replication in infants with acute respiratory diseases are observed, no human pathogenicity for TTV has been fully established.^19^

There is increasing evidence that non-human primates, farm animals including pigs, cows, chickens, sheep, camels, cats, and dogs and wild animals like tupaias (tree shrews) and boars are infected with TTV.^20^^-^^29^ Except for pigs, study of TTV in other farm animals are rare and in small numbers. In chickens in two reports 21 and 117 chickens were tested. Among them, only 4 and 1 cases were positive, respectively.^23^^,^^30^ Since TTV has been detected in different animals, it might be considered as a zoonotic infection. Hence it is possible that the virus can be transmitted from human to animals and vice versa.

As the studies on TTV in chickens was rare and there was no information about the infection of domestic village chickens with TTV, the frequency of the infection in domestic village chickens in different villages in Isfahan (Iran) was investigated.

## Materials and Methods

**Samples. **Blood samples were collected from brachial vein of 50 domestic village chickens from different villages around Isfahan city in Iran. The sera were collected and stored at -20 ˚C till tested.

**DNA extraction. **Viral DNA was extracted using phenol/ chloroform/isoamylalcohol after treatment of 200 μL of serum with 0.5 mg mL^−1^ of proteinase K (Fermentas, St. Leon-Rot, Germany) in the presence of 0.2M NaCl, 0.25% Sodium Dodecyl Sulfate (SDS) for 2 hr at 65 ˚C. The pellet was dried and re-suspended in distilled water or TE (Tris-HCl buffer [10 mM, pH 8.0] containing 1 mM EDTA) solution after precipitation with ethanol and stored at -20 ˚C till tested.

**PCR amplification. **The previously described T801 and T935 primers in a single round of PCR and Set B primers in a nested PCR (Set B forward 1 and Set B reverse 1 primers were used in the first round and Set B forward 2 and Set B reverse 2 primers were used in the second round of nested PCR) were used for amplification of a conserved non-coding region (NCR) of the viral genome.^3^^,^^23^ All the primers described had already been designed utilizing the TTV prototype (TA278) sequence (GenBank accession No. AB008394).^1^^,^^2^ *Taq* DNA polymerase (Roche Diagnostics, Mannheim, Germany) was used. Thermal cycling conditions for PCR with the T801 and T935 primers were as follows: denaturation of 94 ˚C for 5 min followed by 40 cycles of denaturation at 94 ˚C for 20 sec, annealing at 57 ˚C for 25 sec, and extension at 72 ˚C for 30 sec. The amplification was followed by a final extension step at 72 ˚C for 5 min. Thermal cycling conditions for both rounds of nested PCR were as follows: denaturation of 94 ˚C for 5 min followed by 35 cycles of denaturation at 94 ˚C for 20 sec, annealing at 55 ˚C for 30 sec, and extension at 72 ˚C for 30 sec. The amplification was followed by a final extension step at 72 ˚C for 5 min. PCR products were electrophoresed in 2% agarose gel containing ethidium bromide. The expected product sizes with the T801 and T935 primers and Set B primers were 199 bp and 243 bp, respectively. 

**Positive control. **Positive controls used in this investigation, were TTV positive human sera from healthy blood donors in Isfahan, obtained in previous study.^6^

## Results

**Fig. 1:**
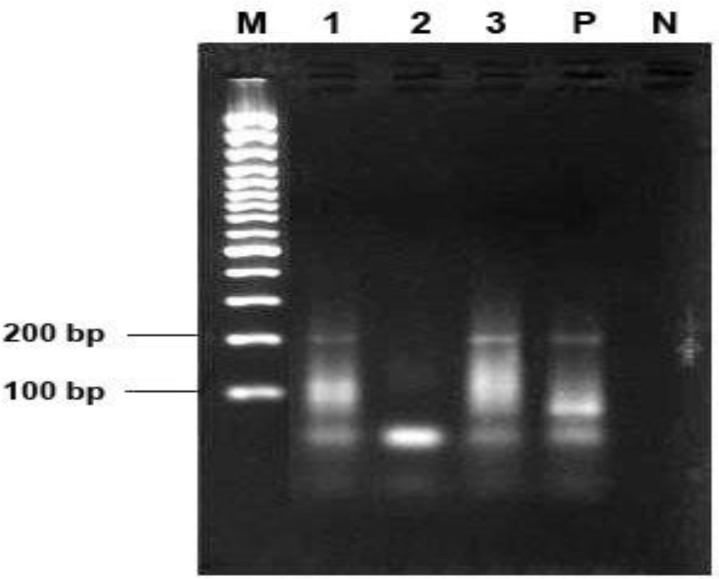
Gel electrophoresis results of single round PCR with the T801 and T935 primers. M = 100 bp marker (Fermentas, St. Leon-Rot, Germany); 1 and 3 = positive samples; 2 = negative sample; P = positive control and N = negative control

**Fig. 2:**
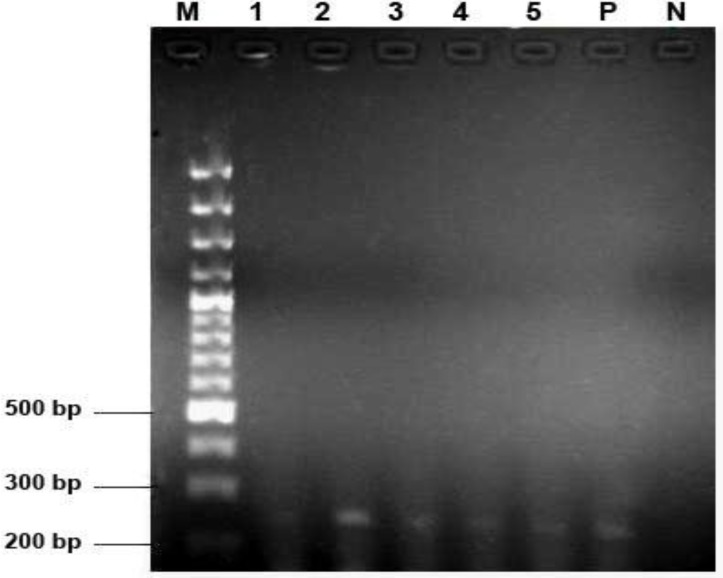
Gel electrophoresis results of the nested PCR with the Set B primers. M = 100 bp marker (Fermentas, St. Leon-Rot, Germany); 1, 2, 3, 4 and 5 = positive samples; P = positive control and N = negative control

In the single round PCR with the T801 and T935 primers and in the nested PCR with Set B primers a 199 bp product (Fig. 1) and a 243 bp product (Fig. 2) were detected respectively. Using T801 and T835 primers TTV or viruses of TTV family were detected in 16 out of 50 sera tested (32%). Fourteen out of the same 50 sera (28%) were positive for TTV using Set B primers. Totally 20 sera were positive using both primers (40%). Ten sera were detected with both sets of primers, six sera with T801 and T935 primers and only four sera were positive using Set B primers for TTV.

## Discussion

The primers used in this study i.e. T801 and T935^3^ and Set B primers^23^ have been designed according to genome of prototype TA278 of the virus (Accession No. AB008394)^1^^,^^2^ for detection of human TTVs. Here we report that they also can detect TTVs of domestic village chickens which are in close contact with human and other domestic farm animals. Due to the detection of TTV in domestic village chickens, it may be possible that TTV can be transmitted from human to village chickens and vice versa.

Evidence for extensive homologous recombination among widely divergent TT viruses is reported.^31^ It is has also been reported that only close types of the virus can recombinate.^32^ There are new evidences that TTVs can be effective on the far related circoviruses i.e. chicken anemia virus (CAV) which is immunosuppressive in new borne chickens, and induces generalized lymphoid atrophy, severe anemia, and increased mortality.^33^ Recently, it is reported that all three major proteins of CAV and TTV have common feature, suggesting close relation between the two viruses and that there is functional similarity in VP3 of TTV and CAV. It is also reported that although complementation of apoptin deficiency by TTV-VP3 cannot be common, since ORF of VP3 is not open in all TTV genotypes and expression of this ORF has not been confirmed in full length TTV clone, replication of chicken anemia virus requires apoptin and is complemented by VP3 of human TTV.^34^ This necessitates more investigations about the prevalence of TTV in chickens and the clinical significance of the co-infection of TTV with CAV and the possibility of their synergism and that which genotypes of TTV might be involved in this process.

Here we reported evidence for the presence of TTV in domestic village chickens that may play a role in transmission of the virus to other domestic animals and human and vice versa. The possible synergism of TTV and CAV should be beard in mind.
